# Rasmussen encephalitis: an older adult presentation?

**DOI:** 10.1590/1980-57642020dn14-040016

**Published:** 2020-12

**Authors:** Ricardo Pires Alvim, Patrick Aguiar, Daniel Kempel Amado, Maria Sheila Guimarães Rocha, Roberta Diehl Rodriguez, Sonia Maria Dozzi Brucki

**Affiliations:** 1 Behavioral and Cognitive Neurology Unit, Department of Neurology, Universidade de São Paulo – São Paulo, SP, Brazil.; 2 Department of Neurology, Hospital Santa Marcelina – São Paulo, SP, Brazil.; 3 Biobank for Aging Studies, LIM-22, Universidade de São Paulo – São Paulo, SP, Brazil.

**Keywords:** dementia, cognitive dysfunction, encephalitis, epilepsy, demência, disfunção cognitiva, encefalite, epilepsia

## Abstract

Rasmussen encephalitis (RE) is a classic disorder in the child age group, and only 10% of cases are described in adults. We bring two proven cases of RE in older adults aged over 55 years.

**Objective:**

To describe the clinical characteristics, progression, diagnostic assessment, neuropathological findings, and treatment of RE in two clinical cases of patients over 55 years of age. Furthermore, we address progressive cognitive decline as an important feature of the RE presentation in older adults in association with focal epilepsy.

**Methods:**

This is a case series from two tertiary hospitals from São Paulo – Brazil. Retrospective data were collected from one case. Results: Two male individuals aged >55 years with clinical presentation of focal epilepsy along with progressive cognitive deterioration.

**Conclusions:**

RE could be considered the cause of progressive cognitive decline in older adults, especially if focal epilepsy is described together with asymmetrical neuroimaging findings.

## RESUMO.

A encefalite de Rasmussen (ER) é um distúrbio clássico da faixa etária infantil e apenas 10% dos casos são descritos em adultos. Trazemos dois casos comprovados de ER em idosos, com idade acima de 55 anos de idade.

**Objetivo:**

Descrever as características clínicas, evolução, avaliação diagnóstica, achados neuropatológicos e tratamento da ER em dois casos clínicos com mais de 55 anos de idade. Além disso, atentar para o declínio cognitivo progressivo como uma característica importante na apresentação ER idosos em associação à epilepsia focal.

**Métodos:**

Série de casos de dois Hospitais Terciários em São Paulo, Brasil. Dados retrospectivos foram coletados de um caso.

**Resultados:**

Dois indivíduos do sexo masculino com idade >55 anos e apresentação clínica de epilepsia focal associada a deterioração cognitiva progressiva.

**Conclusão:**

A ER pode ser considerada a causa do declínio cognitivo progressivo em idosos, especialmente se for descrita epilepsia focal associada a achados assimétricos em neuroimagem.

## INTRODUCTION

In 1958, Rasmussen described the clinical, radiological, and pathological characteristics of a new syndrome in three children (mean age 6.8 years) who had refractory epileptic seizures and progressive neurological impairment.^1^ Rasmussen encephalitis (RE) is a progressive disease characterized by drug-resistant focal epilepsy, progressive hemiplegia, and cognitive decline, with unihemispheric brain atrophy.^2^

Since then, several case reports with similar symptoms have been published. In 2005, the European Association proposed diagnostic criteria for RE.^3^ Most cases involve children and adolescents, but presentation in adulthood is also described in the literature. Adult RE is more prevalent in women (8:1),^4^ but these cases are rare, and the oldest patient was a 54-year-old woman.^5^

In this case report, we provide clinical and neuroimaging data from two patients older than 54 years diagnosed with RE. One of them had a neuropathological evaluation.

## CASE REPORT

### Case 1.

In 2015, a 56-year-old man visited the emergency room with clonic movements in the left arm and oculocephalic deviation to the left, with 24 hours of impaired consciousness. His wife complained about a rapidly progressive cognitive decline in the previous three months, with temporal and spatial disorientation and impaired episodic memory. Additionally, she reported dependence in activities of daily living and behavioral problems, such as apathy and complex visual hallucinations. His history included daily alcohol and tobacco consumption. He was transferred to our service after an episode of *epilepsia partialis continua* and treatment with carbamazepine and phenytoin.

**Figure 1:**
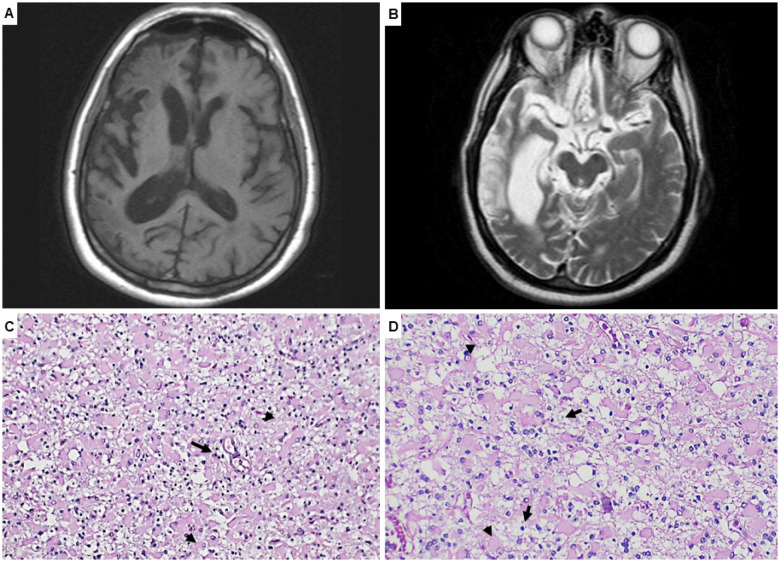
Brain magnetic resonance imaging – T1 axial (A) with evidence of right-side atrophy. T2 axial (B) corroborates the atrophy with temporal hyperintense signal. Histological findings in sections stained with hematoxylin and eosin show grey matter (C) with severe neuronal loss, reactive astrocytes with eosinophilic cytoplasm and tapering processes (arrowhead), and mild perivascular inflammation with few lymphocytes (arrow); white matter (D) presents microcystic cavitation, numerous gemistocytic astrocytes (arrowhead), and histiocytes (arrow). Bars: C – 200 μm; D – 100 μm.

After resolution of status epilepticus, the patient presented bradypsychism and dysarthria, with comprehension of short commands. Neurological examination revealed mild left hemiparesis and spasticity; the Mini-Mental State Examination (MMSE) score was 26 points, the Functional Activities Questionnaire (FAQ — maximum value:30) totaled 21 points, and the Katz Index of Independence in Activities of Daily Living scored 4 points out 6. Electroencephalogram (EEG) demonstrated frequent epileptiform activity with sharp waves in the right temporal region. Cerebral spinal fluid (CSF) showed only hyperproteinorrachia (93 mg/dL). Comprehensive infectious, metabolic, and autoimmune investigation was negative. Brain magnetic resonance imaging (MRI) revealed significant and profound right-side asymmetry, as seen in Figure 1.

One year later, a brain biopsy was performed in the frontal region to help identify the underlying lesion. Besides hematoxylin and eosin staining, the following antibodies were used: CD3, CD20, CD68, GFAP, Ki67, p53, SV40, herpesvirus type 1 and type 2. Microscopically, severe neuronal loss, intense reactive astrocytosis, neuropil vacuolation, microcystic cavitation, histiocytes, and perivascular lymphocytic infiltrate were identified (Figure 1). In addition, abundant macrophages CD68(+), Ki67(-), and few lymphocytes CD3(+), CD20(-) were found. These histological findings are compatible with RE. Furthermore, the presence of severe cortical damage associated with a less prominent inflammatory process suggests a late stage of RE.

Epilepsy was partially controlled with carbamazepine 600 mg/d and lamotrigine 150 mg/d; risperidone 1 mg/d was introduced for behavioral control. He was submitted to monthly immunoglobulin (2 g/kg), with cognitive stabilization. At the last appointment, 3 years later, he scored 24 points in the MMSE and 12 in the FAQ, with a slightly better functional capacity than previously observed.

### Case 2.

A 65-year-old male patient with 10 years of schooling started experiencing symptoms of visual hallucinations and partial motor seizures preceded by screams in 2003 (at the age of 48 years). In the same year, he presented neuropsychiatric symptoms, such as paranoid delusion, depressed mood, and suicidal ideation. Additionally, he had a history of alcohol and tobacco abuse. After two years, his wife described loss of complex activities of daily living and cognitive impairment related to spatial orientation and episodic memory.

At the first neurological examination, he was dysarthric and understood simple commands. Moreover, simultanagnosia and prosopagnosia were detected. He scored 13/30 in the MMSE. Muscle strength was normal; however, he showed bilateral and asymmetric parkinsonism, worse on the left hemibody, and myoclonus on this side. Over the past three years, the patient developed a progressive mild left hemiparesis (Medical Research Council — MRC grade IV/V), with pyramidal signs.

**Figure 2:**
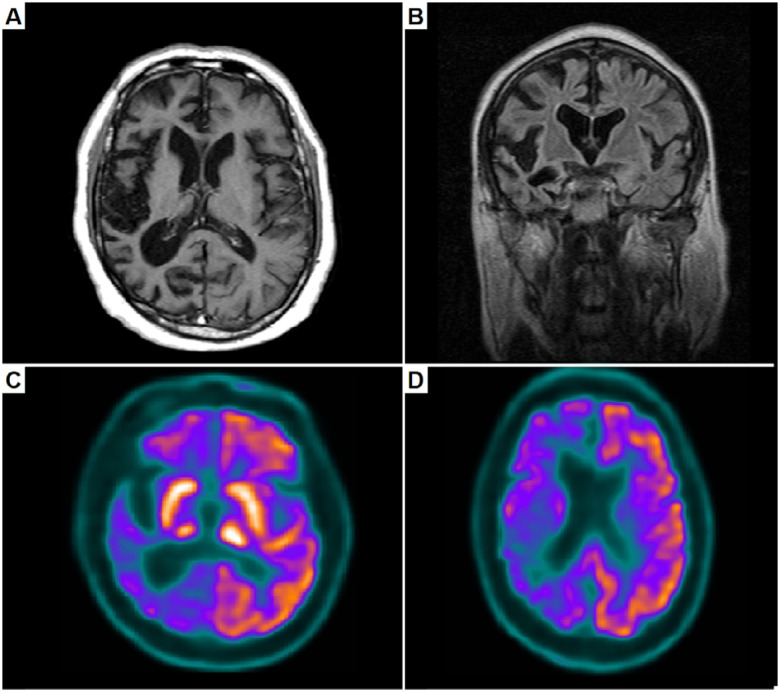
Brain magnetic resonance imaging – T1 axial (A) and coronal (B) reveal right-side atrophy; ^18^F-FDG PET-CT (C and D) shows right-side hypometabolism.

He had no family history of neurological disease. Laboratory screening for dementia was negative, as well as rheumatologic tests and serology for HIV, syphilis, and hepatitis. CSF was normal, and EEG revealed slow waves in the right frontotemporal region, without epileptiform activity. Brain MRI and ^18^F-FDG PET-CT are depicted in Figure 2. MRI angiography was also normal.

Treatment consisted of adjusting antiepileptic drug doses, and corticosteroid pulse therapy was started.

## DISCUSSION

RE is classically described in childhood, and presentations in adolescents and adults correspond to about 10%.^2^ ^,^ ^6^ ^,^ ^7^ On the other hand, descriptions of this disease in older adults are still scarce.^6^ Villani et al.^8^ described a series of seven cases with a mean age of 33.7 years (the oldest at 48 years), and left hemisphere atrophy was the most common. Boyle et al.^4^ reported the case of a 54-year-old female patient with *epilepsia partialis continua*, progressive hemiparesis, and dysfunction of the right parietal lobe, whose pathological examination was compatible with RE. This patient did not respond to immunosuppression.

The current diagnostic criteria proposed by the European consensus involve two possibilities: Part A — partial epilepsy or unilateral cortical deficit, asymmetric EEG (unihemispheric slowing with or without epileptiform activity and unilateral seizure onset), brain MRI with asymmetric atrophy; Part B — *epilepsia partialis continua* or progressive unilateral cortical deficit, brain MRI showing progressive asymmetric cortical or histopathological signs with encephalitis findings mediated by T cells and activated microglia cells.^3^ RE diagnosis is a two-step process: fulfill all three items of Part A criteria; if one item is missing, the patient needs to satisfy two out of three Part B criteria.^3^

The two cases described above meet the criteria, one of them with histopathological diagnosis (part B criteria).^3^ A recent literature review evaluated late RE cases with the following characteristics: a) unclear prodromal stage; b) slower progress of cortical deficits three to nine years after the onset of seizures; c) more favorable outcome than in children. From the beginning, our two cases presented marked progressive cognitive deficits associated with epilepsy, differing somewhat from the data of this review, in which only 37% demonstrated cognitive changes.^4^

Regarding the differential diagnosis of the described cases, we have some highlights. The first case had neuropathological evidence that confirmed our diagnosis. We agree that other inflammatory causes could mimic this presentation, but neuropathological findings are characteristic of RE along with the *epilepsia partialis continua* and asymmetrical MRI described. Thus, this case fulfills the requirements of the European consensus (part B). Regarding the second case, differential diagnosis with other neurodegenerative diseases can be discussed, but we have some comments. First, the age at neurological symptom presentation (48 years old) is not characteristic of any kind of dementia. We point out that he had no family story of dementia, so we could not expect an autosomal dominant genetic mutation that could justify this early clinical manifestation. Second, at 48 years old, the first neurological clinical symptom was focal epilepsy. This clinical presentation is not typical for the initial presentation of neurodegenerative disease. Third, although he had visual hallucinations, this feature was associated with more complex neuropsychiatric symptoms, such as paranoid delusion, suicidal ideation, and alcohol plus tobacco abuse. This behavior is not usually related to Lewy body disease. At the last follow-up in 2019, our patient scored 13/30 in the MMSE (17 years of disease), so we believe that the progression is very atypical for corticobasal syndrome, whose prognosis is 6–8 years.

Finally, the disease mechanism is not yet clearly defined but assumed to be immune-mediated.^2^ ^,^ ^3^ On the other hand, a study has argued that RE does not meet the current criteria for autoimmune encephalitis.^9^ One of our cases was treated with monthly immunoglobulin and showed a response in the partial control of seizures and cognitive status. The second case was started with a high dose of intravenous corticosteroids with no response until now.

Partial epilepsy tends to be drug-resistant in children and adults, but the progression is less catastrophic in older adults.^4^ Nevertheless, they experience a progressive loss of functional capacity secondary to asymmetric atrophy due to the progressive atrophy process.^2^

We described two cases of late presentation of RE through MRI, EEG, ^18^F-FDG PET-CT, and neuropathological analysis. Our data strengthen the view that this disease is not exclusive to childhood and should be considered in older adults with initial manifestations of epilepsy and cognitive decline with loss of functional capacity, together with significant asymmetric atrophy on neuroimaging.

The treatment does not differ from that prescribed to children and involves the control of episodes associated with immunosuppressants; however, hemispherectomy is hardly indicated.
